# Enhancement on the Surface Hydrophobicity and Oleophobicity of an Organosilicon Film by Conformity Deposition and Surface Fluorination Etching

**DOI:** 10.3390/ma11071089

**Published:** 2018-06-26

**Authors:** Zheng-Wen Xu, Yu-Kai Zhang, Tai-Hong Chen, Jin-How Chang, Tsung-Hsin Lee, Pei-Yu Li, Day-Shan Liu

**Affiliations:** 1 Institute of Electro-Optical and Material Science, National Formosa University, 63201 Yunlin, Taiwan; 10576104@gm.nfu.edu.tw (Z.-W.X.); yukai708@hotmail.com.tw (Y.-K.Z.); 10476106@gm.nfu.edu.tw (P.-Y.L.); 2 Additive Manufacturing and Laser Application, Industrial Technology Research Institute, 73445 Tainan, Taiwan; thchen1208@itri.org.tw (T.-H.C.); jinhowchang@itri.org.tw (J.-H.C.); 3 Metal Industries Research & Development Centre, 82151 Kaohsiung, Taiwan; thlee@mail.mirdc.org.tw

**Keywords:** organosilicon film, silver seed layer, conformity deposition, hydrophobicity, oleophobicity, fluorination etching

## Abstract

In this work, the surface morphology of a hydrophobic organosilicon film was modified as it was deposited onto a silver seed layer with nanoparticles. The surface hydrophobicity evaluated by the water contact angle was significantly increased from 100° to 128° originating from the surface of the organosilicon film becoming roughened, and was deeply relevant to the Ag seed layer conform deposition. In addition, the organosilicon film became surface oleophobic and the surface hydrophobicity was improved due to the formation of the inactive C-F chemical on the surface after the carbon tetrafluoride glow discharge etching. The surface hydrophobicity and oleophobicity of the organosilicon film could be further optimized with water and oleic contact angles of about 138° and 61°, respectively, after an adequate fluorination etching.

## 1. Introduction

Anti-fingerprint which keeps surface free of contamination from fingerprints and dirt, as well as making it easier to clean, has been applied on daily-used supplies. More recently, it becomes a critical added-value for application on high-end products such as all smartphones and tablet touch screens to improve aesthetic appearance and reduce maintenance cost. To realize the anti-fingerprint property, products with both surface hydrophobicity and oleophobicity are essential [1,2,3]. Unfortunately, surface repellence to both water and oleic droplets is not available for substrates or devices, since most activate to either water or oleic droplets [4,5,6]. Consequently, surface modification via coating an anti-fingerprint film is demanded to simultaneously achieve surface hydrophobicity and oleophobicity. To realize a surface inactive to water droplet, a lipid film composed of a nonpolar molecule which possesses low surface free energy is commonly deposited, resulting in a surface exhibiting hydrophobicity [7,8,9]. Commonly, the higher is the contact angle to the water droplet, the lower is the surface free energy [10,11]. In our previous work, we demonstrated that an organosilicon film abundant in nonpolar carbon-hydrogen (C-H) related chemical bonds that possess a low surface free energy of about 10 mN/m^2^ exhibited surface hydrophobicity with a water contact angle of about 100° [12,13]. Although the film exhibited hydrophobic surface, the degree of the water angle (WCA) is still far from that of a super-hydrophobic surface (WCA > 150°) required for an anti-fingerprint coating application [1]. 

To approach the film with surface super-hydrophobicity, one method according to the reports announced by Wenzel and Cassie is to roughen the surface to reach a certain degree [14,15]. Recently, regular or random rough surface achieved by the lithographic-patterning, chemical-etching, and layer-by-layer assembly techniques to create a high aspect ratio topographic surface of a silicone- or siloxane-based film has been developed to result in the surface super-hydrophobicity with self-cleaning function [2,16,17,18]. However, although an apparent improvement on the surface hydrophobicity is achievable via roughening the film’s surface, the chemical bond configurations on the film surface mainly composed of the C-H bond showed oleophilicity [6,19]. Consequently, oil and oily substances such as fingerprints and grease are likely to adhere to such an oleophilic surface. Except for engineering the surface roughness, another method to improve the surface hydrophobicity is to introduce carbon-fluorine (C-F) related bonds in the silicone or siloxane matrix by either wet or dry chemical process to minimize the surface energy as well as reduce the chemical interaction to the water droplet. Moreover, because the fluorine atoms in the C-F bond are more electronegative to repel the oleic droplets, it is also reported that the resulting surface is oleophobic with an oleic contact angle (OCA) greater than 60° [17,20,21,22,23]. For instance, Hsieh et al. used one-step sol-gel method to prepare silica-based coatings with fluorine modification. They found that the incorporation of the fluorine atoms to a critical atomic ratio was responsible for a surface with the improved water and oil repellence [17]. Moreover, they also sprayed the copolymer solution of TiO_2_ nanoparticles and perfluoroalkyl onto the silicon substrate to achieve a surface with super-hydrophobicity and oleophobicity [20]. Cansoy et al. studied the effect on the advancing and receding water contact angles of the pattern size and geometry on the silicon wafer realized by deep reactive ion etching where the surface was hydrophobized through vapor phase reaction using the dimethyldichlorosilane solution. They found that both values were correlated with the pattern distance and diameter [18]. Basu et al. sprayed a polydimethylsiloxane (PDMS)-silica nanocomposite coating onto the glass substrate to realize the surface with super-hydrophobicity. They demonstrated that the surface of the PDMS-silica coating became oleophobicity when sprayed with a top-coating using the FAS-13 solution with a low surface energy [21]. However, although the above-mentioned sol-gel and spray methods for preparing silica-based film with the fluorine atoms incorporation are reported to realize the surface with quality hydrophobicity and oleophobicity, these two methods are challenged by the coating uniformity, thickness, and scale-up deposition. Recently, Aminayi et al. successfully applied a FOTS layer with the cotton fabric using nanoparticles deposition in the vapor phase reaction to perform surface ultra-oleophobicity. The nanoparticle roughened fabric surface caused a high static contact angle and a balance dynamic contact angle to the glycerol droplet [22]. In addition, Durret et al. prepared a roughened FEP (fluorinated Ethylene Propylene) flexible film by nano-imprint lithography. They discussed the evolutions of the surface wettability and contact angle hysteresis on the dimension of these nanostructures [23]. In addition to nanostructures prepared using imprint and vapor phase reaction technologies to possibly meet the requirement for depositing functional coatings on large-area substrates, the development of the self-organization of metals such as Au, Ag, and Ni films after a specific annealing treatment is also a promising candidate for achieving three-dimensional nanostructures on the scale-up substrate using vapor phase deposition [24,25,26,27].

In this work, we developed a combination process of the conformity deposition and surface fluorination etching to realize a quality hydrophobic organosilicon film with surface oleophobicity. Surface textures of the organosilicon film were modified and controllable, as it was deposited onto the silver seed layer with specific nanoparticles. The relationship between the surface of the organosilicon film and the Ag seed layer with nanoparticles was investigated by their surface and cross-section observations. Subsequently, carbon tetrafluoride (CF_4_) plasma was employed to etch the roughened organosilicon film. Evidence of the surface fluorination on the organosilicon film etched by the plasma treatment was conducted by the chemical bonds analysis. The C-F related bonds were abundant in the surface of the etched organosilicon film. The plasma treatment was elucidated to be responsible for the optimization of the surface hydrophobicity and the achievement of the surface oleophobicity.

## 2. Material Preparation and Experimental Procedure

A silver seed layer prepared using the sputtering system was deposited onto the clean silicon substrate. The thickness of this seed layer was controlled at various deposition times from 75 to 720 s with a deposition rate about of 4 nm/min. To form the Ag nanoparticles, these silver seed layers were then annealed by a rapid thermal annealing (RTA) treatment at 500 °C for 1 min under nitrogen ambient. After 100 nm-thick organosilicon films were plasma polymerized by the plasma enhanced chemical vapor deposition (PECVD) system at room temperature using the precursor of tetramethylsilane (Si(CH_3_)_4_, TMS) monomer, they were deposited onto the annealed silver seed layers. The deposition pressure, rf power, and gas flow rate of the TMS monomer were fixed at 26 Pa, 70 W, and 75 sccm, respectively. Eventually, the chemical bond configurations on the surface of the organosilicon film were modified by the fluorination etching under the CF_4_ glow discharge for various times (15–60 s). The etching pressure, rf power, temperature, and gas flow rate of the CF_4_ gas were controlled at 78 Pa, 250 W, 70 °C, and 70 sccm, respectively. Another set of the organosilicon films directly deposited onto the silicon substrate with and without the fluorination etching also were prepared as the comparisons.

Film thickness of these hydrophobic organosilicon films was measured using a surface profile system (Dektak 6M, Veeco, New York, NY, USA). Optical transmittance and absorbance of the silver seed layer were conducted by an UV-Vis-NIR spectrophotometer (UVD 3500, Labomed, Inc., Los Angeles, CA, USA). Surface roughness of the silver seed layers and the organosilicon films with and without the fluorination etching process was measured by atomic force microscopy (AFM, DI-3100, Veeco, New York, NY, USA) using the tapping mode. Plane-view and cross-section morphologies of the Ag nanoparticles as well as the organosilicon films deposited onto the Ag seed layers were observed by a field emission scanning electron microscope (FE-SEM, JSM-6700F, JEOL, Tokyo, Japan) operated at 3 kV. The chemical bond nature and functional groups of the films were examined by Fourier transform infrared (FTIR) spectrometry (FT/IR-4100, JASCO, Halifax, NS, Canada). The evolutions of the chemical bond configurations on the film surface with and without the fluorination etching were analyzed by an X-ray photoelectron spectroscope (XPS, ULVAC-PHI, Quantera SXM, Kanagawa, Japan) with monochromatic Al *K*α radiation. Contact angles of the deionized water and hexadecane (C_16_H_34_) liquid droplets on the film surface measured from a contact angle meter (FTA125, Contact Angle Analyzer, First Ten Angstroms, VA, USA) at least 10 times for each sample were employed to evaluate the surface wettability of the film structures to repel water and oil.

## 3. Results and Discussion

**Figure 1:**
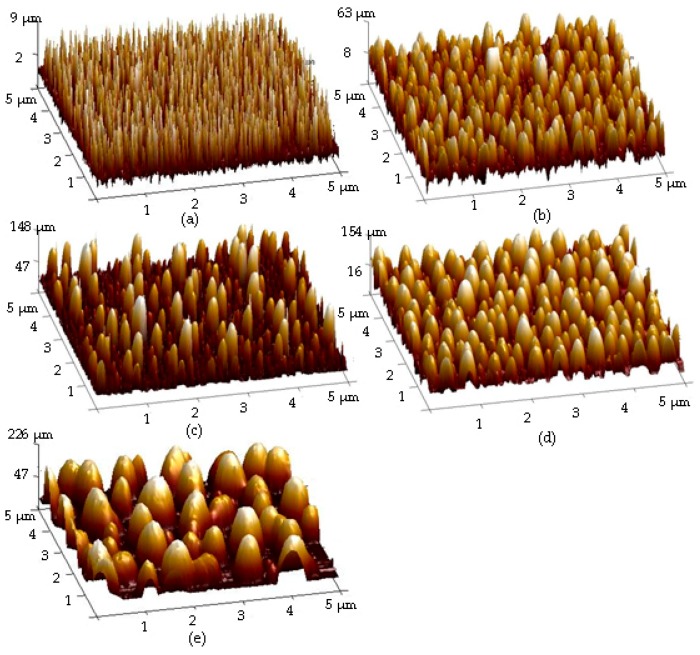
Surface roughness of: (**a**) the as-deposited Ag seed layer; and (**b**–**e**) the Ag seed layer deposited for 75, 150, 480, and 720 s, respectively, and then annealed at 500 °C for 1 min under nitrogen ambient.

**Figure 2:**
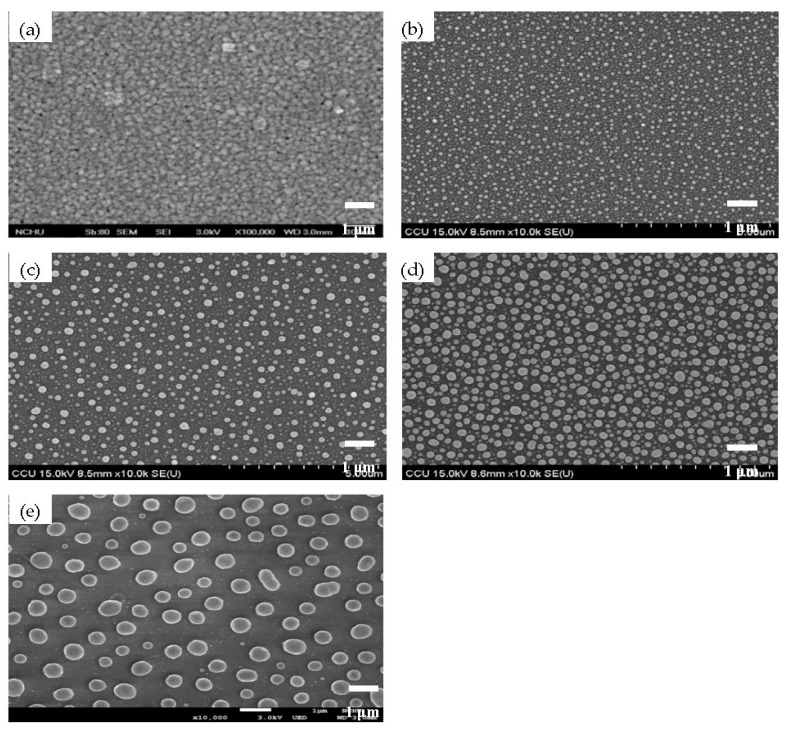
Topographical images by FE-SEM observations of: (**a**) the as-deposited Ag seed layer; and (**b**–**e**) the Ag seed layer deposited for 75, 150, 480, and 720 s, respectively, and then annealed at 500 °C for 1 min under nitrogen ambient.

**Figure 3:**
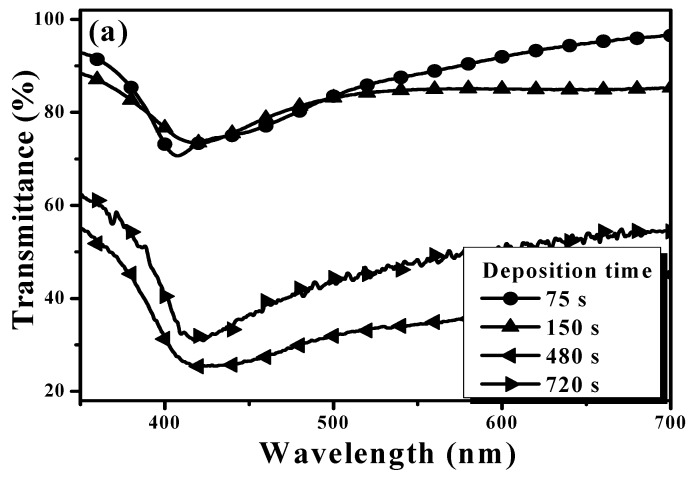

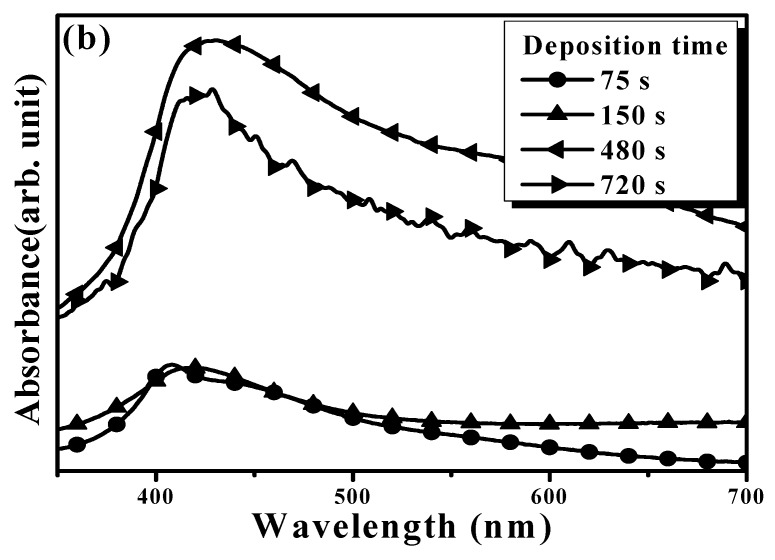
(**a**) Optical transmittance; and (**b**) absorbance spectra of the annealed Ag seed layers as a function of the deposition time.

Surface roughness of the as-deposited Ag seed layer deposited for 150 s and the Ag seed layer deposited for 75, 150, 480, and 720 s and then annealed at 500 °C for 1 min under nitrogen ambient are given in Figure 1a–e, respectively. In Figure 1a, uniform needle-like structures are distributed over the surface of the as-deposited Ag seed layer with a low root-mean-square surface roughness, R_q_, of about 2.0 nm. In contrast to the smooth surface, uneven tip-like structures were observed from the annealed Ag seed layer and the dimension of these structures was found to be increased as the deposition times increased (Figure 1b–e). Table 1 summarizes the surface roughness of these annealed Ag layers. Compared to the as-deposited Ag seed layer, the formation of the tip-like structures distributed over the surface of the annealed Ag seed layer corresponded to a marked increase in the surface roughness (~31.6 nm). In addition, the increase in the dimension of the tip-like structures on the annealed surface also resulted in the increase in the surface roughness. The larger were the tip-like structures observed on the film surface, the higher was the surface roughness measured. A highest surface roughness of about 76.0 nm was thus measured from the annealed Ag seed layer deposited for 720 s (Figure 1e). Figure 2b–e shows the surface morphologies of the annealed Ag seed layer as a function of the deposition time as well as the as-deposited Ag seed layer deposited for 150 s (Figure 2a). The particles distributed over the surface of the as-deposited Ag seed layer (Figure 2a) are densely and tightly packed, whereas round and separated nanoparticles were observed from the surface of the annealed samples. As quoted from the reports, the formation of these isolated Ag nanoparticles was ascribed to the compressive stress induced by the annealing treatment and the Ostwald ripening mechanism [28,29]. It was also evident that the size of these nanoparticles was increased with the deposition time of the Ag seed layer increasing. The mean diameters of these round nanoparticles calculated by the software ImageJ analysis are summarized in Table 1 [30]. The mean size of the nanoparticles for the annealed Ag seed layer deposited for 75 s was about 72 nm, while the thick Ag seed layer deposited for 720 s resulted in a large nanoparticle size of about 232 nm after the RTA treatment. The optical transmittance and absorbance spectra of the annealed Ag seed layer as a function of the deposition time are depicted in Figure 3a,b. As can be seen in Figure 3a, the opaque Ag seed layers with the transmittance of nearly 0% (not shown in the figure) became transparent after being processed by the RTA treatment as a consequence of the incident light leaked through the nanoparticle boundaries. The average transmittance at visible wavelengths (400~700 nm), T_ave_, as summarized in Table 1, decreased as the deposition time of the Ag seed layer increased. Average transmittances higher than 80% were obtained from the annealed Ag seed layers deposited for 75 and 150 s, while the thick Ag seed layers deposited for 480 s annealed by the RTA treatment corresponded to an average transmittance lower than 40%. Compared to the annealed Ag seed layer deposited for 480 s, the slight increase in the average transmittance of the annealed Ag seed layer deposited for 720 s (~46%) was ascribed to the reduction in the surface coverage of the Ag nanoparticles, as listed in Table 1. In addition, according to the literature [31,32,33], the obvious peak that appeared in the absorbance spectra of these annealed samples, as shown in Figure 3b, was in connection with the localized surface plasmon (LSP) resonance emerged from the formation of the Ag nanoparticles. Consistent with previous reports [34,35], the increase in the mean size of the Ag nanoparticles analyzed by the software ImageJ, as shown in Table 1, also corresponded to the red-shift of the absorbance peak in Figure 3b. 

**Figure 4:**
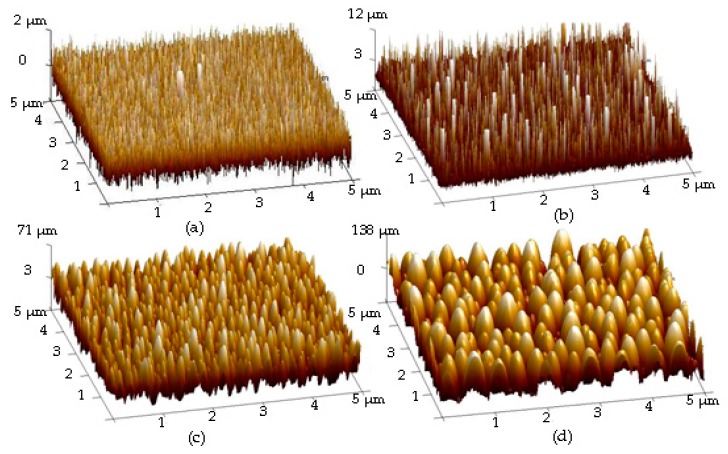
Surface Roughness of: (**a**) the organosilicon film directly deposited onto the silicon substrate; and (**b**) the film deposited onto the as-deposited Ag seed layer; as well as the films deposited onto the annealed Ag seed layers deposited at for: (**c**) 150 s; and (**d**) 480 s.

**Figure 5:**
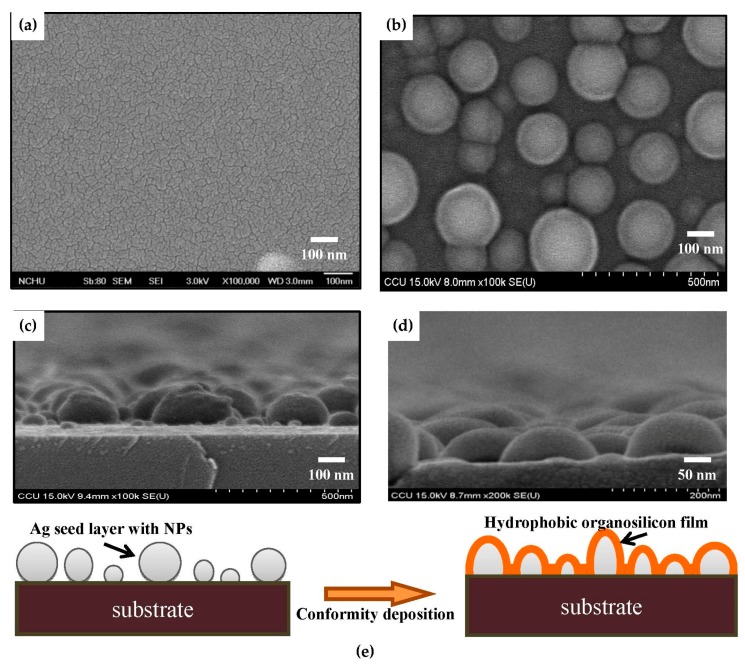
Topographical images by FE-SEM observations of the organosilicon films deposited onto: (**a**) the silicon substrate; and (**b**) the annealed Ag seed layer. Cross-section images by FE-SEM observations of: (**c**) the annealed Ag seed layer; and (**d**) the organosilicon film deposited onto the annealed Ag seed layer. (**e**) A schematic representation of the conformity deposition.

According to the above experiments, size-dependent nanoparticles caused the evolution of the surface roughness, which were realized by controlling the deposition time of the Ag seed layer followed by an RTA treatment. Figure 4c,d shows the surface roughness of the hydrophobic organosilicon films deposited onto the annealed Ag seed layer deposited for 150 and 480 s, respectively. The organosilicon films directly deposited onto the silicon substrate and onto the as-deposited Ag seed layer surface, respectively, are also given in Figure 4a,b for comparison. The organosilicon film directly deposited onto the silicon substrate was quite smooth with a very low surface roughness of 0.4 nm. As the organosilicon film deposited onto the as-deposited Ag seed layer (Figure 4b), needle-like structures similar to those that appeared on the as-deposited Ag surface, as shown in Figure 1a, were observed to lead to a slight increase in the surface roughness (~1.9 nm). It was also noted that these needle-like structures distributed over the organosilicon film surface evolved into tip-like shape as it was deposited onto the annealed Ag seed layer, and also corresponded to a high surface roughness of about 18.1 nm, as shown in Figure 4c. Similar to the size-dependent of the Ag nanoparticles, the size of the tip-like structures on the organosilicon film surface also increased as the deposition time of the annealed Ag seed layer increased. A significant increase in the surface roughness (~49.8 nm) was measured from the organosilicon film deposited onto the annealed Ag seed layer deposited for 480 s (Figure 4d). Surface morphologies of the organosilicon films deposited onto the silicon substrate and the annealed Ag seed layer surface are shown in Figure 5a,b. The surface morphology of the organosilicon film seemed to be smooth and densely packed with obvious channels, as it was directly deposited onto the silicon substrate. By contrast, separated particles with various dimensions were observed on the surface morphology of the organosilicon film deposited onto the annealed Ag seed layer. Cross-section morphologies of the annealed Ag seed layer and the organosilicon film deposited onto this annealed seed layer are shown in Figure 5c,d. Semi-spherical structures appear in Figure 5d that are similar to the nanostructures observed in Figure 5c, further confirming that the organosilicon film grew confirmty on the Ag nanoparticles. Accordingly, significant increase in the surface roughness of the organosilicon film that was comparable to that of the Ag seed layer with the nanoparticles was achievable from this conform deposition, as presented in Figure 5e.

**Figure 6:**
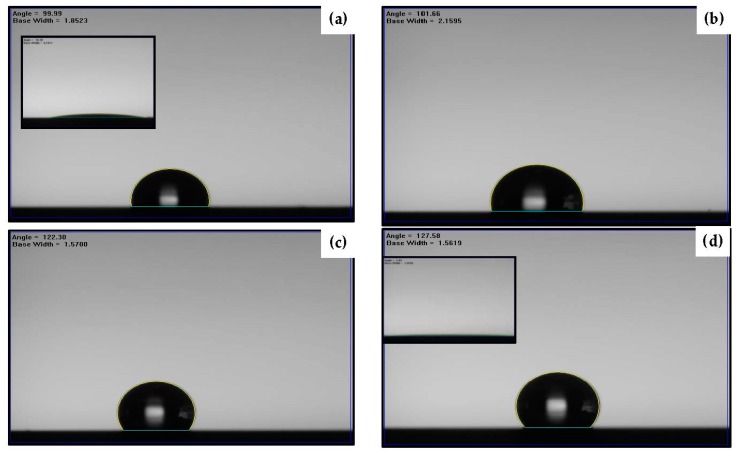
Water contact angle of: (**a**) the organosilicon film directly deposited onto the silicon substrate; and (**b**) the film deposited onto the as-deposited Ag seed layer; as well as the films deposited onto the annealed Ag seed layers deposited at for: (**c**) 150 s; and (**d**) 480 s (the inset figures show the correspondent oleic contact angle).

Surface hydrophobicity determined by the water droplets contacted to the organosilicon films deposited onto the silicon substrate and onto the Ag seed layers with and without an RTA treatment are shown in Figure 6a–d, respectively. The measured water contact angles and the corresponding surface roughness are summarized in Table 2. The surface hydrophilicity of the silicon substrate with a water contact angle less than 40° became hydrophobic with a water contact angle of about 100° as it was modified by coating the organosilicon film (Figure 6a). A slight increase in the water contact angle of about 102° was measured from the surface of the organosilicon film deposited onto the as-deposited Ag seed layer (Figure 6b), which concurrently exhibited only a small increase in the surface roughness as compared to the organosilicon film directly deposited onto the silicon substrate. The roughened surface of the organosilicon film when it was deposited onto the annealed Ag seed layer with nanoparticles, as observed in Figure 4c, resulted in a large water contact angle of about 122° (Figure 6c), revealing that the significant improvement on the hydrophobic property was achievable from the film deposited onto the patterned Ag underlayer. The surface hydrophobicity could be further optimized to a water contact angle of 128° as the organosilicon film deposited onto the annealed Ag seed layer deposited for 480 s (Figure 6d). As can be seen in Table 2, the evolution on the water contact angle of the organosilicon film was seen to be deeply relevant to the increase in the film’s surface roughness. A simple model proposed by Wenzel equation, as represented in following equation, can be referred to characterize the surface wettability of the organosilicon film surface affected by its surface roughness [36]:

(1) $$cos\Theta^{*}=\gamma cos\Theta$$

where *γ* is the roughness factor defined as the ratio between the actual surface area of a rough surface to the projected area. *Θ* and Θ^*^ are the contact angles of the liquid droplets on the flat and patterned surface, respectively. According to the relationship between the surface roughness and the hydrophobicity of the organosilicon film, one can achieve a quality hydrophobic surface simply by using the conformity deposition process which was demonstrated as the PECVD-deposited organosilicon film grew conformably to the patterned Ag seed layer with size-controlled nanoparticles prepared by an RTA treatment through the above-mentioned surface and cross-section observations.

**Figure 7:**
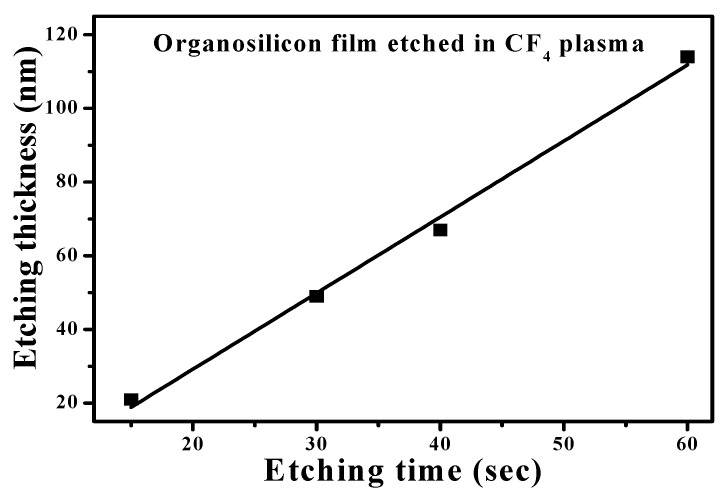
Etching thickness of the organosilicon film treated by the CF_4_ plasma as a function of the etching time.

**Figure 8:**
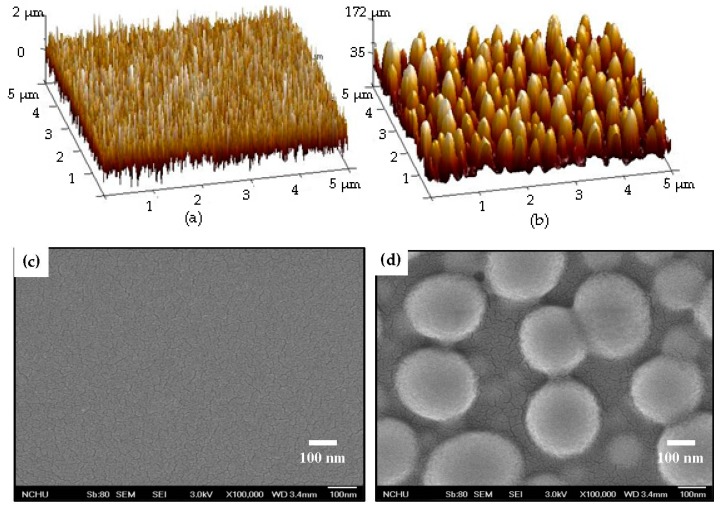
Surface roughness and morphologies of the CF_4_ plasma etching for 40 s on the organosilicon film deposited onto: (**a**,**c**) the silicon substrate; and (**b**,**d**) the as-deposited Ag seed layer.

**Figure 9:**
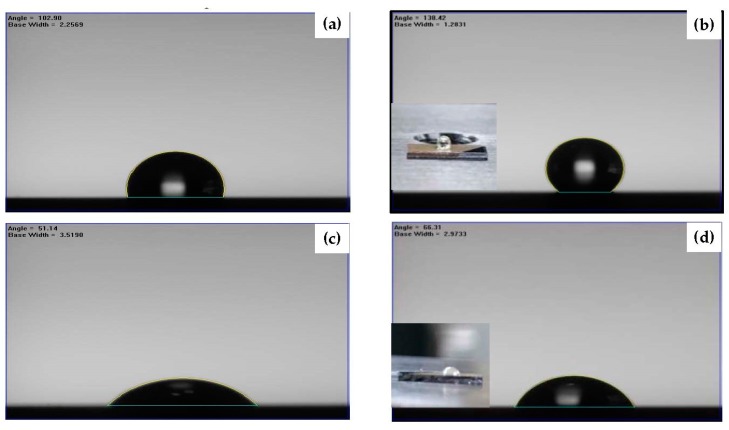
Water contact angles of: (**a**) the organosilicon film directly deposited onto the silicon substrate; and (**b**) the film deposited onto the annealed Ag seed layer after etching by the CF_4_ plasma for 40 s; and (**c**,**d**) the corresponding oleic contact angles (the inset images show the water and oleic droplets on the film surface).

However, though the specific area of the organosilicon film had been successfully enlarged as it was deposited onto the Ag nanoparticles and showed less activated to the water droplet to result in a large water contact angle, this surface still was very sensitive to the oleic droplet. As can be seen in the inset figures of Figure 6a,d, both samples showed oleophilic surface with the oleic contact angles of about 11° and 3°, respectively. To active the surface of the organosilicon film to repel the oleic droplet, surface modification via engineering the chemical bond configuration was carried out by etching the film in the CF_4_ glow discharge. Figure 7 illustrates the etching thickness of the organosilicon film in the CF_4_ plasma ambient as a function of the etching times. The etching thickness of the organosilicon film was linearly correlated to the time of the film exposed to the CF_4_ plasma ambient. As the etching time reached 60 s, an average etching thickness of about 118 nm, exceeding 100 nm, was obtained. The surface roughness of the organosilicon films deposited onto the silicon substrate and onto the annealed Ag seed layer after etching by the CF_4_ plasma for 40 s is shown in Figure 8a,b, respectively. The surface roughness of the organosilicon films directly deposited onto the silicon substrate and onto the annealed Ag seed layer after treating by the CF_4_ plasma etching was only slightly increase to about 0.6 and 51.7 nm, respectively, as compared to the samples without the CF_4_ plasma treatment (Figure 4a,d). The correspondent surface morphologies of the etched organosilicon film investigated from the SEM observations, as shown in Figure 8c,d, are also very similar to those of the un-etched samples (Figure 5a,b). This revealed that the physical ion-bombardment to cause the change in the surface textures was less important than the chemical reaction to result in the bond-reconstruction on the organosilicon film induced by the CF_4_ plasma etching. Water contact angles of these two organosilicon films etched by the CF_4_ plasma for 40 s are presented in Figure 9a,b, respectively. The surface hydrophobicity of the organosilicon film directly deposition onto the silicon substrate evaluated from the water contact angle showed slight increase from 100° to 102° as it was treated by the CF_4_ plasma etching for 40 s, whereas the water contact angle of the organosilicon film deposited onto the annealed Ag seed layer was increased more significantly after the CF_4_ plasma etching. In addition, the surface of these etched organosilicon films became oleophobic with oleic contact angles of about 51° and 68°, as shown in Figure 9c,d, respectively. Table 3 summarizes the evolutions on the water and oleic contact angles of the organosilicon film directly deposited onto the silicon substrate and onto the annealed Ag seed layer after etching by the CF_4_ plasma for various times. Although the increase in the surface roughness of the flat organosilicon film directly deposited onto the silicon substrate etched by the CF_4_ plasma was very limited, its water contact (~103°) was comparable to that of the organosilicon film deposited onto the as-deposited Ag seed layer which performed a higher surface roughness of about 1.9 nm, indicating the surface modification on the chemical bonds achieved by the CF_4_ plasma etching was also facilitated to optimize the film’s surface hydrophobicity. Moreover, the increase in the water contact angle of the etched organosilicon film was more evident as it was deposited onto the annealed Ag seed layer. Such textured surface of the organosilicon surface deposited onto the annealed Ag seed layer which might allow larger specific area to be chemically modified than that of the flat organosilicon film deposited onto the silicon substrate was likely to be responsible for the apparent increase in the water contact. The water contact angle listed in Table 3 was increased with the film surface etched by the CF_4_ plasma increasing, and a largest water contact angle of about 138° was measured from the organosilicon film surface etched by the CF_4_ plasma for 40 s. However, because the etching thickness exceeded the thickness of the organosilicon film (~100 nm), a drastic decrease in the water contact angle correlating to the surface wettability of the annealed Ag seed layer was thus measured (~70°). In regard to the surface activity to the oleic droplets shown in Table 3, the surface of the organosilicon film became oleophobic with an oleic contact angle higher than 50° as it was chemically modified by the CF_4_ plasma. In addition, similar to the evolutions of the water contact angles of the organosilicon films etched by the CF_4_ plasma, the evolutions of the oleic contact angle were also strongly correlated to the films’ surface roughness. It was also noted that the surface of the etched organosilicon film again evolved into surface oleophilicity with an oleic contact angle of about 7°, a degree close to the oleic droplet on the annealed Ag seed layer (~3.1°), as it was etched for 60 s in the CF_4_ plasma.

**Figure 10:**
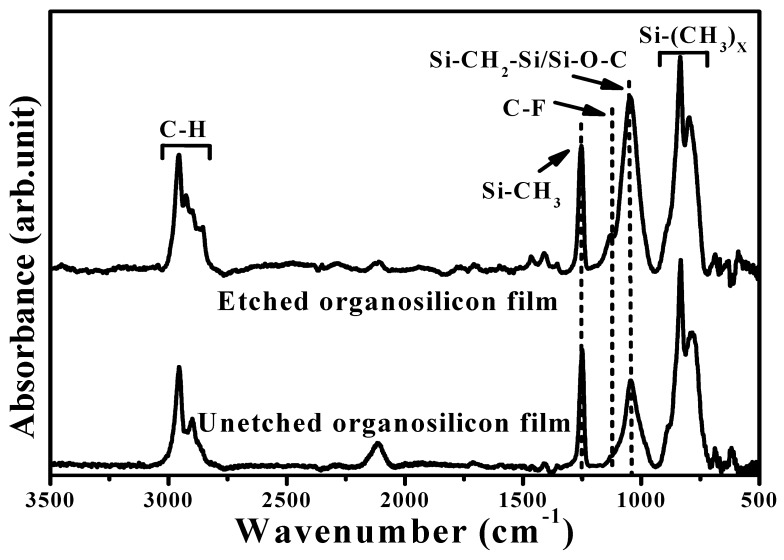
FTIR spectra of the organosilicon film before and after the CF_4_ plasma etching for 40 s.

**Figure 11:**
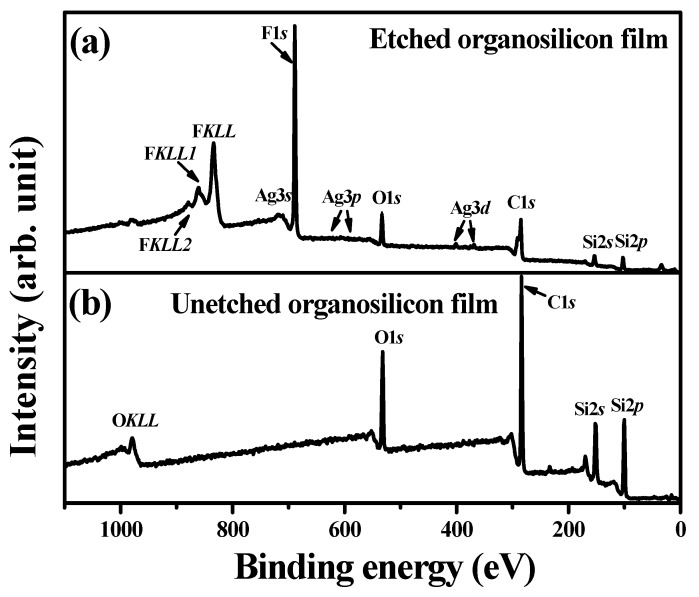
XPS survey spectra taken on the surface of the organosilicon film (**a**) with and (**b**) without the CF_4_ plasma etching.

**Figure 12:**
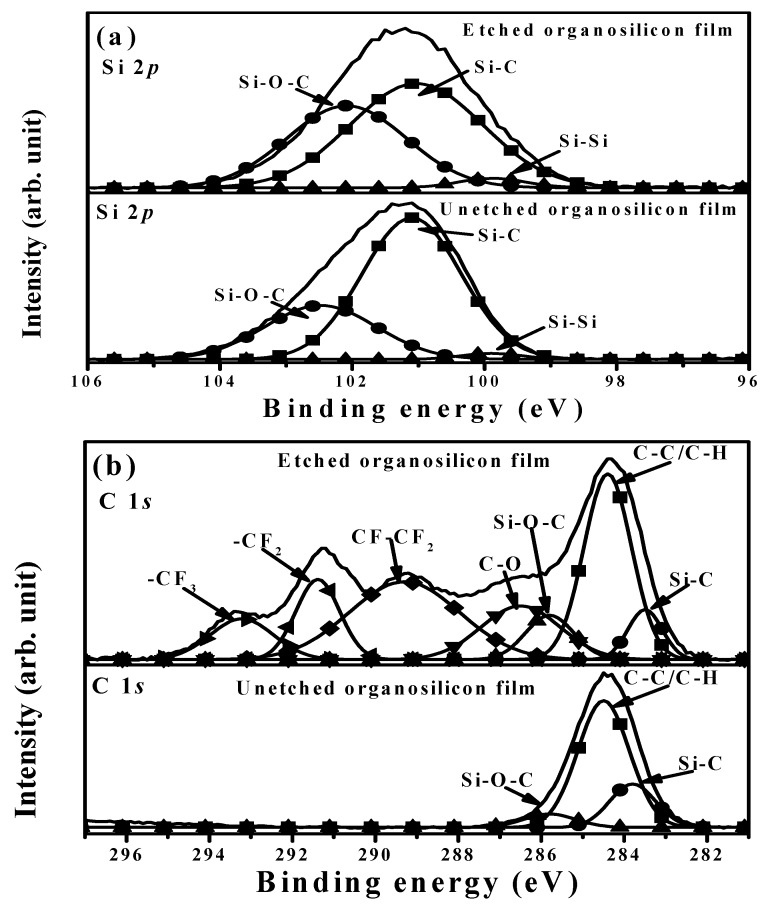
XPS spectra of the (**a**) Si 2*p* and (**b**) C 1*s* core levels for the organosilicon films with and without the CF_4_ plasma etching.

**Figure 13:**
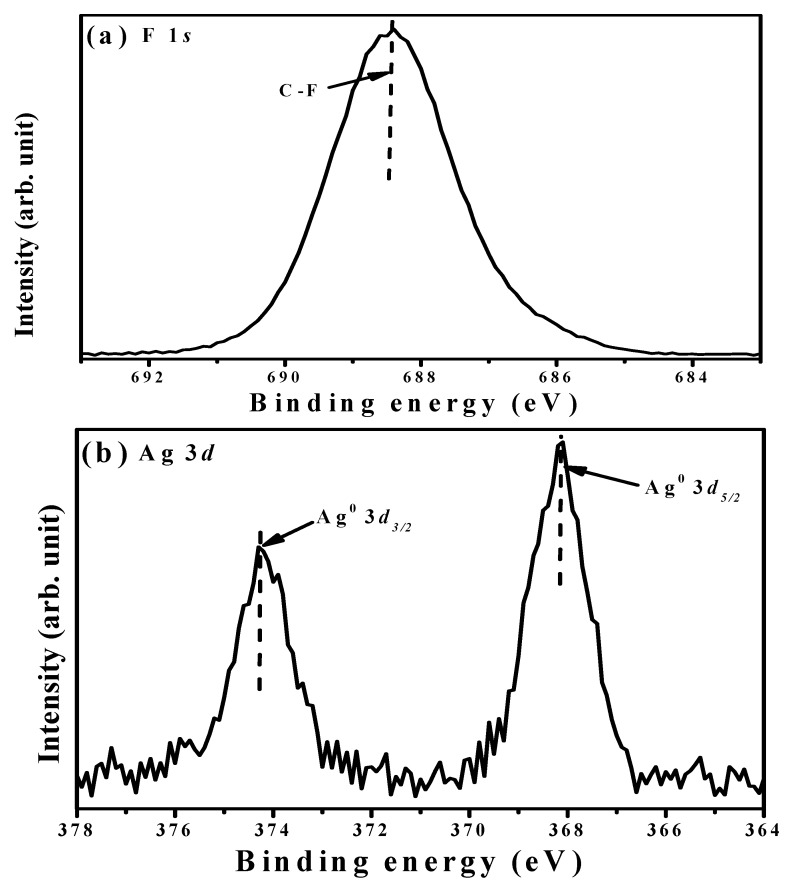
XPS spectra of the (**a**) F 2*s* and (**b**) Ag 3*d* core levels for the organosilicon films etched by the CF_4_ plasma.

FTIR spectra of the organosilicon film before and after the CF_4_ plasma etching for 40 s are illustrates in Figure 10. The FTIR spectrum of the organosilicon film etched by the CF_4_ plasma was almost identical to that of the film without the CF_4_ plasma etching. Four absorbance peaks predominated over these two FTIR spectra. The broad absorbance band with peaks at about 797 and 835 cm^−1^, and a sharp peak at about 1253 cm^−1^ were denoted as the deformation of the Si-C rocking vibration and symmetric bending in the Si-CH_3_ groups, while another broad signal located at high wavenumbers of about 2899 and 2955 cm^−1^ was characterized as the hydrophobic bond associated with the C-H bond in the symmetrical and asymmetrical stretching of CH_3_ groups [37,38,39,40]. The absorbance peak at about 1047 cm^−1^ was assigned as the networks of the Si-CH_2_-Si bond overlapped with the Si-O stretching vibration mode in the Si-O-C bond [13,41]. In contrast to the organosilicon film without the CF_4_ plasma etching, a shoulder at around 1128 cm^−1^ related to the absorbance that emerged from the C-F chemical bond, as observed in the etched organosilicon film [42,43,44]. Figure 11a,b shows the XPS survey spectra taken on the surface of the organosilicon film with and without the CF_4_ plasma etching. The surface of the organosilicon film without the CF_4_ etching was dominated by the elements of C, Si, Si, and O. By contrast, the intensity of the C, Si, and O signal was apparently decreased and the element of F became the dominant signal over the surface of the organosilicon film etched by the CF_4_ plasma, showing evidence of the introduced fluorine atoms on the film surface. In addition, very weak Ag signal could be observed from the etched organosilicon film. The high-resolution XPS spectra of the Si 2*p* and C 1*s* core levels for the organosilicon films with and without the CF_4_ plasma etching are presented in Figure 12a,b, respectively. In Figure 12a, the surface of the organosilicon film both with and without the CF_4_ plasma etching showed a peak at around 101.2 eV. This peak could be deconvoluted into three major peaks at 99.9, 101.0, and 102.1 eV, which were in turn characterized as the chemical bond states of Si-Si, Si-C, and Si-O-C [45,46,47]. The surface of the organosilicon film without the plasma etching was basically constructed from the cross-linking Si-C bond. When the film etched by the CF_4_ plasma, the ion-bombardment damage induced by the CF_4_ plasma caused the breaking of the Si-C chemical bond seemed to be responsible for the obvious reduction in the relative intense. The C 1*s* core levels on the surface of the unetched organosilicon film shown in Figure 12b only presented an intense C 1*s* signal at about 284.3 eV. This peak was composed of three C-related bonds at about 283.8, 284.5, and 285.8 eV, respectively, which could be assigned as the Si-C, C-C/C-H, and Si-O-C chemical bonds [48,49]. At the surface of the organosilicon film etched by the CF_4_ plasma, an apparent tail extending to the high binding energy other than the main C 1*s* peak at 284.3 eV was measured. According to the literature [50,51,52], this tail could be deconvoluted into four specific peaks associated with the C-O, CF-CF_2_, -CF_2_, and -CF_3_ chemical bonds at about 286.5, 289.3, 291.4, and 293.2 eV, respectively. The formation of these fluorine-related chemical bonds was strong evidence of the achievement on the surface fluorination using the CF_4_ plasma etching. In addition, since these fluorinated groups was helpful to lower the surface energy of the organosilicon film, especially for the -CF_3_ bond which was reported to possess the lowest surface energy among these groups [20,53]. Thus, this surface presented quality repellence to water and oleic droplets. Consequently, the surface of the organosilicon film abundant in the C-F functional groups exhibited a superior water contact angle to the film only constructed from the Si-C/Si-O-C groups and also evolved surface oleophobicity when it was treated by the CF_4_ plasma etching. Figure 13a,b, respectively, depicts the core levels of the F 1*s* and Ag 3*d* signal measured on the surface of the etched organosilicon film. As can be seen in Figure 13a, the fluorinated surface of the organosilicon film using the CF_4_ plasma treatment shows an obvious peak at about 688.4 eV that was assigned as C-F chemical bond [54,55]. In addition, the peaks at approximately 368.1 and 374.3 eV in Figure 13b were identified as the chemical state of the metallic Ag (denoted as Ag^0^ 3*d*_5/2_ and Ag^0^ 3*d*_3/2_, respectively), in accordance with previous reports [56,57]. The metallic Ag signal likely emerged from the annealed Ag seed layer. According to the investigations on the FTIR and XPS measurements, the fluorine atoms were successfully introduced into the surface of the organosilicon film using the CF_4_ plasma etching to form the nonpolar C-F chemical bond, thereby further optimizing the film’s surface energy to improve the surface hydrophobicity as well as achieve an oleophobic surface.

## 4. Conclusions

Surface textures of a hydrophobic organosilicon film were simply modified by the Ag seed layer. Size-dependent nanoparticles of the Ag seed layer annealed at 500 °C for 1 min under nitrogen ambient were obtained from the seed layer deposited for different time. The subsequently deposited organosilicon film using the PECVD system was found to grow conformably to the Ag seed layer. Such a roughened surface of the organosilicon film similar to the Ag seed layer controlled by the conformity deposition realized the improvement on the resulting surface hydrophobicity. The water contact angle of the organosilicon film deposited onto the annealed Ag seed layer with the nanoparticles at an average size of 181 ± 97 nm was apparently increase to 128° as compared to the organosilicon film directly deposited onto the flat silicon substrate (~100°). Moreover, the chemical bond states on the surface of the organosilicon film were modified using CF_4_ plasma etching. Although little change on the surface roughness was measured from the organosilicon film etched by the CF_4_ plasma, the surface hydrophobicity was further optimized, especially for the film with surface textures that allowed more specific area for the surface fluorination. The reason for the enhanced surface hydrophobicity of the etched organosilicon film was ascribed to the appearance of the C-F related functional groups on the film surface formed during CF_4_ plasma etching. Such etched surface abundant in the C-F bonds also evolved from oleophilic into oleophobic because of the C-F bonds, thus was inactive to the oleic droplet. Accordingly, an organosilicon film with water and oleic contact angles of about 138° and 60°, respectively, was achieved from the film conformably deposited onto the annealed Ag seed layer with the nanoparticles, and then etched by the CF_4_ plasma treatment for 40 s. This functional film, exhibiting both surface hydrophobicity and oleophobicity, is a promising candidate for a quality anti-fingerprint coating.

## Figures and Tables

**Table 1:** Surface roughness (R_q_), mean particle size, average transmittance (T_ave_), surface coverage, and absorbance peak of the Ag seed layer deposited for various time after annealed at 500 °C for 1 min under nitrogen ambient.

	Sample	Ag Deposition Time (s)			
Parameter		75	150	480	720
R_q_ (nm)		18.2	31.6	52.8	76.0
Mean particle size (nm)		72 ± 44	84 ± 66	181 ± 97	232 ± 138
T_avg_ (%)		86	82	35	46
Surface coverage (%)		20.4	22.9	41.3	28.1
Absorbance peak (nm)		411	417	437	438

**Table 2:** Surface roughness, water contact angle, and oleic contact angle of the organosilicon film directly deposited onto the silicon substrate and deposited onto the as-deposited and annealed Ag seed layer.

Sample	R_q_ (nm)	WCA (°)	OCA (°)
Organosilicon on silicon substrate	0.4	100 ± 0.2	11 ± 1.1
Organosilicon on as-deposited Ag seed layer	1.9	103 ± 0.6	10 ± 0.4
Organosilicon on annealed Ag seed layer (150 s)	18.1	122 ± 0.5	7 ± 0.5
Organosilicon on annealed Ag seed layer (480 s)	49.8	128 ± 0.8	3 ± 0.3

**Table 3:** Water contact angle and oleic contact angle of the organosilicon film directly deposited onto the silicon substrate and deposited onto the annealed Ag seed layer after etching by the CF4 plasma for various time.

Sample		CF4 Plasma Etching Time (s)				
		0	15	30	40	60
Organosilicon on silicon substrate	WCA (^o^)	100 ± 0.2	104 ± 0.3	103 ± 0.2	103 ± 0.3	103 ± 0.4
	OCA (^o^)	11 ± 1.1	50 ± 1.4	51 ± 1.0	51 ± 1.6	50 ± 0.8
Organosilicon on annealed Ag seed layer	WCA (^o^)	128 ± 0.8	132 ± 0.9	135 ± 0.8	138 ± 0.6	70 ± 0.7
	OCA (^o^)	3 ± 0.3	60 ± 0.6	62 ± 0.9	66 ± 0.7	7 ± 0.2

## References

[1] Uhlmann P., Frenzel R., Voit B., Mock U., Szyszka B., Schmidt B., Ondratschek D., Gochermann J., Roths K. (2007). Research agenda surface technology: Future demands for research in the field of coatings materials. Prog. Org. Coat..

[2] Wu L.Y.L., Nigian S.K., Chen Z., Xuan D.T.T. (2011). Quantitative test method for evaluation of anti-fingerprint property of coated surface. Appl. Surf. Sci..

[3] Yin L., Yang J., Tang Y., Chen L., Liu C., Tang H., Li C. (2014). Mechanical durability of superhydrophobic and oleophobic copper meshes. Appl. Surf. Sci..

[4] Li H., Wang J., Yang L., Song Y. (2008). Superoleophilic and superhydrophobic inverse opals for oil sensors. Adv. Funct. Mater..

[5] Huang Y., Zhou J., Su B., Shi L., Wang J., Chen S., Wang L., Zi J., Song Y., Jiang L. (2012). Collodial Photonic Crystals with narrow stopbands assembled from low-adhesive superhydrophobic substrates. J. Am. Chem. Soc..

[6] Owen M.J. (2017). Silicone hydrophobocity and oleophilicity. Silicon.

[7] Kwok D.Y., Neumann A.W. (1999). Contact angle measurement and contact angle interpretation. Adv. Colloid Interface Sci..

[8] Zhu L., Hao G., Chen Y., Chen Y. (2012). Investigation on hydrophobic film from a hydrophobic powder. Appl. Surf. Sci..

[9] Xu K., Hu J., Jiang X., Meng W., Lan B., Shu L. (2018). Anti-icing performance of hydrophobic silicone-acrylate resin coatings on wind blades. Materials.

[10] Kujawa J., Al-Gharabli S., Kujawski W., Knozowska K. (2017). Molecular grafting of fluorinated and nanofluorinated Alkylsiloxanes on various ceramic membrane surface for the removal of volatile organic compounds applying vacuum membrane distillation. ACS Appl. Mater. Interface.

[11] Al-Gharabli S., Hamad E., Saket M., El-Rub Z.A., Arafat H., Kujawski W., Kujawa J. (2018). Advanced material-ordered nanotubular ceramic membranes covalently capped with single-wall carbon nanotubes. Materials.

[12] Wu C.Y., Chen W.C., Liu D.S. (2008). Surface modification layer deposition on flexibility substrates by plasma-enhanced chemical vapour deposition using tertamethylsilane-oxygen gas mixture. J. Phys. D Appl. Phys..

[13] Liu D.S., Wu C.Y. (2010). Adhesion enhancement of hard coatings deposited on flexible plastic substrates using an interfacial buffer layer. J. Phys. D Appl. Phys..

[14] Öner D., McCarthy T.J. (2000). Ultrahydrophobic surface. Effects of topography length scales on wettability. Langmuir.

[15] Shirtcliffe N.J., Mchale G., Newton M.I., Perry C.C. (2003). Intrinsically superhydrophobic organosilica sol-gel foams. Langmir.

[16] Koch K., Bhushan B., Barthlott W. (2009). Multifunctional surface structures of plants: An inspiration for biomimetics. Prog. Mater. Sci..

[17] Hsieh C.T., Wu F.L., Chen W.Y. (2009). Super water- and oil-repellences form silica-based nanocoatings. Surf. Coat. Technol..

[18] Cansoy C.E., Erbil H.Y., Akar O., Akin T. (2011). Effect of pattern size and geometry on the use of Cassie-Baxter equation for superhydrophobic surfaces. Colloid Surf. A-Physicochem. Eng. Asp..

[19] Tian D., Song Y., Jiang L. (2013). Patterning of controllable surface wettability for printing techniques. Chem. Soc. Rev..

[20] Hsieh C.T., Chen J.M., Kuo R.R., Lin T.S., Wu C.F. (2005). Influence of surface roughness on water- and oil-repellent surfaces coated with nanoparticles. Appl. Surf. Sci..

[21] Basu B.J., Kumar V.D., Anandan C. (2012). Surface studies on superhydrophobic and oleophobic polydimethylsiloxane–silica nanocomposite coating system. Appl. Surf. Sci..

[22] Aminayi P., Abidi N. (2015). Ultra-oleophobic cotton fabric prepared using molecular and nanoparticle vapor deposition methods. Surf. Coat. Technol..

[23] Durrent J., Frolet N., Gourgon C. (2016). Hydrophobicity and anti-icing performances of nanoimprinted and roughened fluoropolymers film under overcooled temperature. Microelectron. Eng..

[24] Gingery D., Bühlmann P. (2008). Formation of gold nanoparticles on multiwalled carbon nanotubes by thermal evaporation. Carbon.

[25] Franc J., Bastl Z. (2008). Nickel evaporation in high vacuum and formation of nickel oxide nanoparticles on highly oriented pyrolytic graphite. X-ray photoelectron spectroscopy an atomic force microscopy study. Thin Solid Films.

[26] Abou El-Nour K.M.M., Eftaiha A., Al-Warthan A., Ammar R.A.A. (2010). Synthesis and applications of silver nanoparticles. Arab. J. Chem..

[27] Gromov D.G., Pavlova L.M., Savitsky A.I., Yu T.A. (2015). Nucleation and growth of Ag nanoparticles on amorphous carbon surface from vapor phase formed by vacuum evaporation. Appl. Phys. A.

[28] Kang C.Y., Chao C.H., Shiu S.C. (2007). Formation of self-organized platinum nanoparticles and their microphotoluminescence enhancement in the visible light region. J. Appl. Phys..

[29] Schmiitt J., Hajiw S., Lecchi A., Degrouard J., Salonen A., Impéror-Clerc M., Pansu B. (2016). Formation of superlattices of gold nanoparticles using Ostwald ripening in emulsions: transition for *fcc* to *bcc* structure. J. Phys. Chem. B.

[30] Igathinathane C., Pordesimo L.O., Columbus E.P., Batchelor W.D., Methuku S.R. (2008). Shape identification and particles size distribution from basic shape parameters using Image. J. Comput. Electron. Agric..

[31] Maier S.A., Atwater H.A. (2005). Plasmonics: Localization and guiding of electromagnetic energy in metal/dielectric structures. J. Appl. Phys..

[32] Willets K.A., Wan Duyne R.P. (2007). Localized surface plasmon resonance spectroscopy and sensing. Annu. Rev. Phys. Chem..

[33] Xu W.F., Chin C.C., Hung D.W., Wei P.K. (2013). Transparent electrode for organic solar cells using multilayer structures with nanoporous silver film. Sol. Energy Mater. Sol. Cells.

[34] Zhang S.G., Zhang X.W., Yin Z.G., Wang J.X., Si F.T., Gao H.L., Dong J.J., Liu X. (2012). Optimization of electroluminescence from n-ZnO/AlN/p-GaN light-emitting diodes by tailoring Ag localizer surface plasmon. J. Appl. Phys..

[35] Mogensen K.B., Kneipp K. (2014). Size-dependent shifts of plasmon resonance in silver nanoparticle films using controlled dissolution: Monitoring the onset of the surface screening effects. J. Phys. Chem. C.

[36] Wenzel R.N. (1936). Resistance of solid surface to wetting by water. Ind. Eng. Chem..

[37] Hong B.S., Han J.H., Kim S.T., Cho Y.J., Park M.S., Dolukhanyan T., Sung C. (1999). Endurable water-repellent glass for automobiles. Thin Solid Films.

[38] Tan I.H., da Silva M.L.P., Demarquette N.R. (2001). Paper surface modification by plasma deposition of double layers of organic silicon compounds. J. Mater. Chem..

[39] Teshima K., Sugimura H., Inoue Y., Takai O. (2003). Gas barrier performance of surface-modified silica films with grafted organosilane molecules. Langmuir.

[40] Kim J.D., Lee K.Y., Kim K.Y., Sugimura H., Takai O., Wu Y., Inoue Y. (2003). Characteristics and high water-repellency of a-C:H films deposited by r.f. PECVD. Surf. Coat. Technol..

[41] Wu C.Y., Liao R.M., Lai L.W., Jeng M.S., Liu D.S. (2012). Organosilicon/silicon oxide gas barrier structure encapsulated flexible plastic substrate by using plasma-enhanced chemical vapor deposition. Surf. Coat. Technol..

[42] Nakagawa T., Hiwatashi T. (2003). Water-repellent thin films from mixtures of fluoroalkylmethoxysilane and bis-(trialkoxysilyl)alkanes of various carbon-chain lengths using the sol–gel method and the fluoroalkylmethoxysilane dispersion mechanism. J. Non-Cryst. Solids.

[43] Kang G.S., Ko H.J., Choi C.K. (2003). Chemical bond structure of a-C:F films with a low dielectric constant deposited by using CH4/CF4 ICPCVD. J. Korean Phys. Soc..

[44] Chen G., Zhang J., Yang S. (2008). Fabrication of hydrophobic fluorinated amorphous carbon thin films by an electrochemical route. Electrochem. Commun..

[45] Choi W.K., Ong T.Y., Tan L.S., Loh F.C., Tan K.L. (1998). Infrared and x-ray photoelectron spectroscopy studies of as-prepared and furnace-annealed radio-frequency sputtered amorphous silicon carbide films. J. Appl. Phys..

[46] Brinkmann M., Chan V.Z.H., Thomas E.L., Lee V.Y., Miller D., Hadjichristidis N., Avgeropoulos A. (2001). Room-temperature synthesis of a-SiO_2_ thin films by UV-assisted ozonolysis of a polymer precursor. Chem. Mater..

[47] Trey S.M., Sidenvall P., Alavi K., Ståhlberg D., Johansson M. (2009). Dual cure (UV/thermal) primers for composite substrates—Effect of surface treatment and primer composition on adhesion. Prog. Org. Coat..

[48] Khung Y.L., Nghlim S.H., Meda L., Narducci D. (2014). Preferential formation of Si-O-C over Si-C linkage upon thermal grafting on hydrogen-terminated silicon (111). Chemistry.

[49] David L., Bhandavat R., Barrera U., Singh G. (2016). Silicon oxycarbide glass-graphene composite paper electrode for long cycle lithium-ion batteries. Nat. Commun..

[50] Ferraria A.M., da Silva J.D.L., do Rego A.M.B. (2003). XPS studied of directly fluorinated HDPE: Problem and solution. Polymer.

[51] Wang C., Lai P.C., Syu S.H., Leu J. (2011). Effects of CF_4_ plasma treatment on the moisture uptake, diffusion, and WVTR of poly(ethylene terephthalate) flexible films. Surf. Coat. Technol..

[52] Lakshmi R.V., Bharathidasan T., Bera P., Basu B.J. (2012). Fabrication of superhydrophobic and oleophobic sol–gel nanocomposite coating. Surf. Coat. Technol..

[53] True J.E., Thomas T.D., Winter R.W., Gard G.L. (2003). Electronegativities from core-ionization energies: Electronegativities of SF5 and CF3. Inorg. Chem..

[54] Dai Y., Cai S., Wu L., Yang W., Xie J., Wen W., Zheng J.C., Zhu Y. (2014). Surface modified CFx cathode material for ultrafast discharge and high energy density. J. Mater. Chem. A.

[55] Li Y., Veith G.M., Browning K.I., Chen J., Hensley D.K., Paranthaman M.P., Dai S., Sun X.G. (2017). Lithium malonatoborate additives enabled stable cycling of 5 V lithium metal and lithium ion batteries. Nano Energy.

[56] Ma J., Guo X., Zhang Y., Ge H. (2014). Catalytic performance of TiO_2_@Ag composites prepared by modified photodeposition method. Chem. Eng. J..

[57] Liu F.C., Li J.Y., Chen T.H., Chang C.H., Lee C.T., Hsiao W.H., Liu D.S. (2017). Effect of Silver Dopants on the ZnO Thin Films Prepared by a Radio Frequency Magnetron Co-Sputtering System. Materials.

